# Impact of REAC Regenerative Endogenous Bioelectrical Cell Reprogramming on MCF7 Breast Cancer Cells

**DOI:** 10.3390/jpm13061019

**Published:** 2023-06-20

**Authors:** Vania Fontani, Sara Cruciani, Sara Santaniello, Salvatore Rinaldi, Margherita Maioli

**Affiliations:** 1 Department of Regenerative Medicine, Rinaldi Fontani Institute, 50144 Florence, Italy; vfontani@irf.it; 2 Department of Adaptive Neuro Psycho Physio Pathology and Neuro Psycho Physical Optimization, Rinaldi Fontani Institute, 50144 Florence, Italy; 3 Research Department, Rinaldi Fontani Foundation, 50144 Florence, Italy; sara.cruciani@outlook.com (S.C.); sara.santaniello@gmail.com (S.S.); 4 Department of Biomedical Sciences, University of Sassari, 07100 Sassari, Italy; mmaioli@uniss.it (M.M.)

**Keywords:** MCF7, cell proliferation, cellular mechanisms, senescence, breast adenocarcinoma, endogenous bioelectric activity, endogenous bioelectric fields, radio electric asymmetric conveyer

## Abstract

Human breast adenocarcinoma is a form of cancer which has the tendency to metastasize to other tissues, including bones, lungs, brain, and liver. Several chemotherapeutic drugs are used to treat breast tumors. Their combination is used to simultaneously target different mechanisms involved in cell replication. Radio electric asymmetric conveyer (REAC) technology is an innovative technology, used both in vitro and in vivo, to induce cell reprogramming and counteract senescence processes. Within this context, we treated MCF-7 cells with a regenerative (RGN) REAC treatment for a period ranging between 3 and 7 days. We then analyzed cell viability by trypan blue assays and gene and protein expression by real time-qPCR and confocal microscope, respectively. We also detected the levels of the main proteins involved in tumor progression, DKK1 and SFRP1, by ELISA and cell senescence by β-galactosidase tests. Our results showed the ability of REAC RGN to counteract MCF-7 proliferation, probably inducing autophagy via the upregulation of Beclin-1 and LC3-I, and the modulation of specific tumorigenic biomarkers, such as DKK1 and SPFR1. Our results could suggest the application of the REAC RGN in future in vivo experiments, as an aid for the therapeutic strategies usually applied for breast cancer treatment.

## 1. Introduction

Endogenous bioelectricity as a factor influencing cell development and organization is an exciting emerging discipline [1]. All cells are bioelectric entities that produce endogenous bioelectric fields (EBF) and signals [2]. EBF and signals originate from ionic flows. Due to an asymmetrical distribution of ions across cell membranes, ions constantly flow through cell membranes, thanks to the ion channels and the action of pumps and exchangers [3]. These ion fluxes produce currents, and these produce EBF, so variations in ionic fluxes affect the formation of EBF, thus influencing the normal development of cells [3]. 

EBF and electrical signals are fundamental regulators of cellular behavior in a wide variety of cell types [4]. Bioelectric signals modulate membrane voltage gradients (V_mem_) [5,6] and activate many intracellular signaling pathways, directly influencing cell proliferation, migration, and differentiation, during either embryonic development or tissue repairing [7]. Bioelectric signals can control the rate of mitosis, which is closely related to differentiation, as plastic cells tend to proliferate more than most terminally differentiated somatic cells [8]. Undifferentiated mesenchymal stem cells are depolarized, and increase their polarization during adipogenic or osteogenic differentiation [9]. This is also true for cancer cells [3,10]. It has recently been shown that changes in EBF due to ion flux modifications can affect tumor cell proliferation and metastatic invasion [11,12]. The role of the physiology of EBF and signaling in a variety of cancer types [13] is increasingly emerging [14,15]. EBF and signaling are a source of non-genetic information that is currently increasingly considered an important factor in the onset of cancer [16]. Moreover, the influence of EBF and signaling has also been highlighted in the processes that favor the development of solid tumor metastasis [15,17]. All of this has allowed the conception of the hypothesis that endogenous bioelectric manipulation could become a future therapeutic strategy to fight cancer [18]. Within this context, different studies have been carried out to verify how bioelectric modifications could affect the fate of various tumor cell lines. One of these is the MCF7 breast cancer cell line [19,20].

In this study, we used the Radio Electric Asymmetric Conveyer (REAC) technology for endogenous bioelectric manipulation, with the tissue optimization regenerative (TO-RGN) treatment [21,22,23,24], aimed at reorganizing the endogenous bioelectric activity at the cellular level, to verify if we were able to determine a reprogramming effect on the MCF7. 

We have previously demonstrated the ability of REAC TO-RGN endogenous bioelectric manipulation to reprogram other tumor cell lines, as PC12, derived from a pheochromocytoma of the rat adrenal medulla [21], and to counteract the evolution of myelodysplastic syndrome modulating bone marrow-derived stem cell senescence [25]. 

The REAC general functioning principle is based on determining an endogenous electrical potential difference inside the organism, which is able to release the alterations of charges, which alter the ionic fluxes and therefore the EBF, promoting the phenomena of differentiation, proliferation, migration, and changes in gene expression profiles [21,22,23,24,26,27,28]. Based on these considerations, in this study, we analyzed a set of genes that are involved in senescence and autophagy. These genes include octamer-binding transcription factor 4 (Oct-4), sex determining region Y-box 2 (SOX2), and homeobox protein NANOG (NANOG), which are transcription factors that play a crucial role in maintaining stem cell pluripotency [29]. Additionally, the study looked at cellular myelocytomatosis oncogene (c-Myc), a transcription factor that regulates cell growth and division [30]. We have also investigated RNA silencing suppressor p19 ARF (p19ARF), a gene that helps regulate the cell cycle and prevent uncontrolled cell growth. The cyclin-dependent kinase inhibitor 1 (p21) gene, which is involved in regulating the cell cycle [31], was also included in the analysis. Furthermore, the study examined tumor suppressor protein p53 (p53), which plays a key role in preventing cancer by inducing cell cycle arrest or apoptosis [32]. Finally, the study analyzed B-cell lymphoma 2 (Bcl-2) and heat shock protein 70 (HSP70), two genes that are involved in regulating apoptosis and cellular stress responses [33].

## 2. Materials and Methods

### 2.1. Radio Electric Asymmetric Conveyer (REAC) Technology

Cellular processes rely on the asymmetry of charges for optimal expression of cellular components and functions [34] at the genetic and epigenetic levels [7]. However, various factors can disrupt this asymmetry, leading to compromised cellular functions, including genetic and epigenetic modifications [5,35]. The Radio Electric Asymmetric Conveyer (REAC) technology has been developed to restore the correct asymmetry of intracellular charges and progressively recover altered cellular functions, including negative epigenetic modifications [21,25,28].

The REAC technology platform restores the correct endogenous bioelectric activity (EBA) by conveying asymmetric ion flows. This is achieved through the interaction of the weak radioelectric fields emitted by the device with charge alterations that block the generation of the correct gradients necessary for ionic flows and the endogenous bioelectrical activity. The REAC technology’s unique capability to interact with cells and tissues at the molecular level through asymmetric conveyance of ion flows enables it to interact with charge alterations without depth limits, making it a highly effective tool for restoring EBA in cells and tissues.

Restoration of EBA is crucial for proper cellular function and the expression of genetic and epigenetic processes. The REAC technology’s ability to restore the correct asymmetry of intracellular charges offers potential benefits, such as the restoration of cellular functions, optimization of cellular metabolism, and promotion of regenerative [24,36,37,38]. and reparative [39,40] processes.

### 2.2. REAC TO-RGN Treatment

The REAC device applied in the study was the BENE 110 device (ASMED, Florence, Italy), with treatment parameters for REAC TO-RGN [21,22,23,24,25,26,27] predetermined by the manufacturer and not subject to modification by the operator.

### 2.3. Cell Culturing

The MCF7 breast cancer cell line was acquired from ATCC (Manassas, VA, USA). The cells were cultured in a medium composed of Dulbecco’s modified Eagle’s medium (DMEM, Thermo Fisher Scientific, Waltham, MA, USA), 10% fetal bovine serum (FBS, Thermo Fisher Scientific, USA), 200 mM L-glutamine (Thermo Fisher Scientific) and 200 U/mL penicillin−0.1 mg/mL streptomycin (Thermo Fisher Scientific, USA). The REAC-RGN treatment is a pre-programmed treatment. Cells were cultured in the absence or presence of REAC- RGN treatment for different time points. 

A group of cells was exposed to REAC RGN treatment for 72 h and then put in culture for additional 4 days (a total of 7 days). 

A second group of cells was exposed to REAC RGN treatment for 72 h and then put in culture for additional 7 days (a total of 10 days).

**Figure 1:**
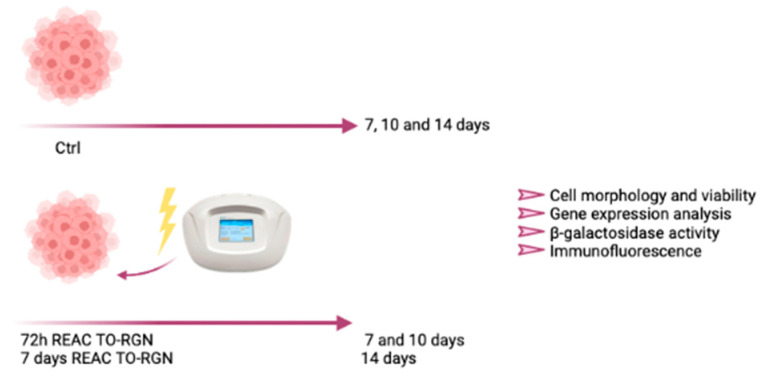
Cell culturing conditions. Created with BioRender.com.

A third group of cells was exposed to REAC RGN treatment for 7 days and then put in culture for additional 7 days (a total of 14 days). For each group, the untreated controls were represented by cells cultured in the growing medium alone (Figure 1). The experiments were performed two times with three technical replicates for each sample.

### 2.4. Trypan Blue Exclusion Test of Cell Viability

The trypan blue dye exclusion test was used to evaluate the number of viable cells cultured in the presence or absence of REAC RGN treatment. The cell suspension obtained from each sample was mixed with 0.4% dye solution (Thermo Fisher Scientific, USA) in a proportion of 1:1, then counted using a LUNA-II^TM^ Automated Cell Counter to obtain information about the cell viability and cell size distributions. The percentage of vital cells was calculated as the number of positive cells divided by the total number of counted cells (mean ± SD) [41].

### 2.5. Gene Expression Analysis by Real-Time PCR

RNA extraction was performed using the Trizol reagent (Life Technologies, Carlsbad, CA, USA) according to manufacturer instructions, in cells either exposed or not to electromagnetic fields. Approximately 1 μg of total isolated mRNA was reverse-transcribed into cDNA using the Superscript Vilo cDNA synthesis kit (Thermo Fisher Scientific, USA), for a final volume of 20 μL. A quantitative polymerase chain reaction was performed in triplicate according to the protocol specified in the Platinum^®^ Quantitative PCR SuperMix-UDG Kit (Thermo Fisher Scientific, USA) using a CFX-96 Thermal Cycler (Bio-Rad) (Applied Biosystems). The total volume of each reaction was 25 μL, composed of 2X SuperMix with SYBR Green I, 0.1 μM of each primer (Table 1), and 3 μL cDNA generated from 1 μg of the total RNA template. 

The standard qRT-PCR conditions were 50 °C for 2 min, 95 °C for 2 min, then cycled at 95 °C for 15 s, 55–59 °C for 30 s and 60 °C for 1 min for 40 cycles. Each experiment included a distilled water control. The target Ct values were normalized on hGAPDH, considered as a reference gene, while the mRNA levels of MCF7 cultured in the presence or absence of REAC RGN treatment were expressed as the fold of change (2^−∆∆Ct^) relative to the mRNA levels observed in the MCF7 at time 0, before starting treatment. The genes analyzed were octamer-binding transcription factor 4 (Oct-4); sex determining region Y-box 2 (SOX2) and homeobox protein NANOG (NANOG); cellular myelocytomatosis oncogene (c-Myc); RNA silencing suppressor p19 ARF (p19ARF); cyclin-dependent kinase inhibitor 1 (p21); tumor suppressor protein p53 (p53); B-cell lymphoma 2 (Bcl-2) and heat shock protein 70 (HSP70). All primers were obtained from Invitrogen (Thermo Fisher Scientific, USA).

### 2.6. Senescence-Associated β-Galactosidase Staining

Cell senescence was evaluated in MCF7 either exposed or not to REAC RGN treatment for either 72 h or 7 d using Senescence-associated (SA) β-Galactosidase Staining Kit (Cell Signaling Technology, Euroclone, Milan, Italy). After treatment, cells were fixed and processed according to the manufacturer’s instructions. Detection of SA-β-Galactosidase activity was performed by inverted light microscope (10 x magnification in bright field). The number of positively blue-stained cells was calculated as the percentage of total number of cells, using an image software analysis (ImageJ, version 1.8.0, National Institutes of Health, Bethesda, MD, USA). 

### 2.7. ELISA Assay

The concentrations of human secreted frizzled-related protein 1 (SFRP1) and human Dickkopf-related protein 1 (DKK1) were determined using the human secreted frizzled-related protein 1 (SFRP1) ELISA Kit (Cusabio, Flarebio Biotech LLC) and the human DKK1 (Dickkopf-related protein 1) ELISA Kit (Elabscience Biotechnology Co., Ltd, Houston, TX, USA), respectively. Cell culture supernatants were collected after 7, 10, and 14 days from MCF7 cultured under the above-described conditions. According to the manufacturer instructions, 100 μL of each sample was incubated in a pre-treated plate for 2 h at 37 °C. After sample removal, a detection antibody was added in each well for 1 h at 37 °C and then washed three times by a washing buffer. Streptavidin-HRP solution was incubated for 1 h at 37 °C. The antibody was then washed three times, and the liquid substrate incubated for 30 min at 37 °C. Stop solution was added at the end of the incubation time, and color development was measured at 450 nm using a plate reader (Akribis Scientific, Common Farm, Frog Ln, Knutsford, UK). Standard curves were prepared according to manufacturer’s instructions. 

### 2.8. Immunostaining

MCF7 (8.0 × 10^4^ cells/well in 8-well chamber slides) were cultured for 7, 10 or 14 days in the above-described conditions and fixed with 4% of paraformaldehyde (Sigma Aldrich Chemie GmbH, Germany) for 30 min at room temperature. After permeabilization by 0.1% Triton X-100 (Thermo Fisher Scientific, Grand Island, NY, USA)-PBS, cells were washed three times for 5 min in PBS. After washing, cells were incubated with 3 % Bovine Serum Albumin (BSA)—0.1% Triton X-100 in PBS (Thermo Fisher Scientific, Grand Island, NY, USA) for 1 h, then exposed overnight at 4 °C to the primary anti-rabbit polyclonal antibodies directed against LCIII and Beclin-1, (Sigma-Aldrich, Germany). Finally, cells were washed three times in PBS for 10 min and stained at 37 °C for 1 h in the dark with the fluorescence-conjugated goat anti rabbit IgG secondary antibody (Life Technologies, USA). Nuclei were labelled with 1 μg/mL 4,6-diamidino-2-phenylindole (DAPI) (Thermo Fisher Scientific, Grand Island, NY, USA). All microscopy analyses were performed with a confocal microscope (TCS SP5, Leica, Nussloch, Germany).

### 2.9. Statistical Analysis

Statistical analysis was performed using GraphPad Prism 9.0 software (GraphPad, San Diego, CA, USA). Wilcoxon signed-rank test was used to evaluate the congruity of the observed set, while a Kruskal–Wallis rank sum was used to assess the values found in the different observation topics, assuming a *p* value ≤ 0.05 as statistically significant. The experiments were performed two times, with three technical replicates for each sample.

## 3. Results

### 3.1. REAC TO-RGN Treatment Inhibits Cell Proliferation and Viability

**Figure 2:**
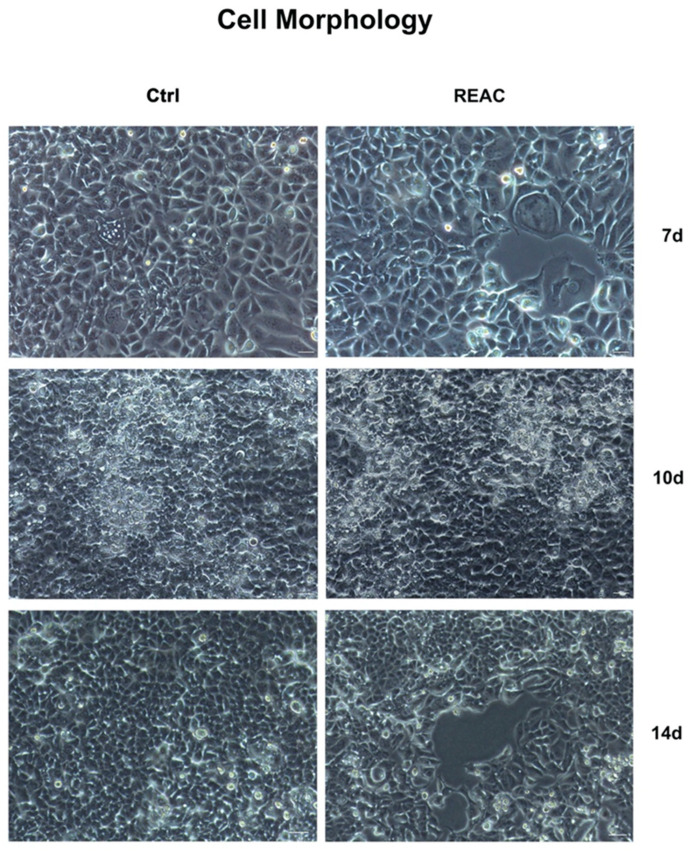
Optical microscope analysis of MCF7 morphology either exposed or not to REAC TO-RGN treatment. Cells were cultured for a total of 7, 10 or 14 days, as described above. Figures of cells exposed to REAC TO-RGN treatment were compared to control untreated cells (Ctrl). Magnification 10×. Scale bar = 100 µm.

MCF7 morphology was evaluated by optical microscopy after 7, 10 and 14 days in culture. Figure 2 shows changes in cell number and confluency after exposure to the REAC TO-RGN treatment, as compared to control untreated cells (Ctrl).

**Figure 3:**
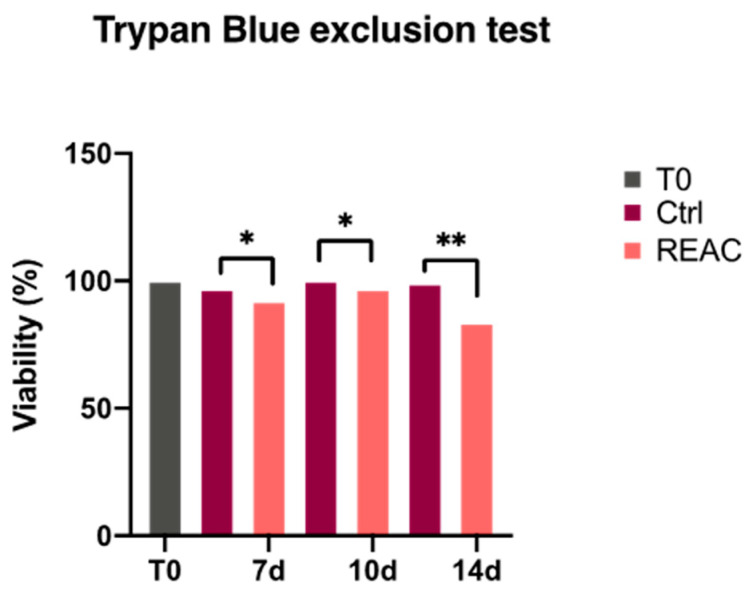
MCF7 viability after REAC TO-RGN treatment. The cells were cultured in the absence or presence of REAC TO-RGN treatment, and counted using an automatic cell counter after 7, 10 and 14 days. The percentage of vital cells was calculated as the number of positive cells divided by the total number of counted cells (mean ± SD) (* *p* ≤ 0.05); (** *p* ≤ 0.01).

These results were further inferred by trypan blue test (Figure 3). The MCF7 were collected and counted using an automatic cell counter. The REAC TO-RGN treatment significantly decreased the MCF7 viability and proliferation at each time point analyzed, as compared to control untreated cells (Ctrl). 

Further, cell dimension was reduced after REAC TO-RGN treatment (Table 2).

### 3.2. REAC TO-RGN Treatment Counteract Cell Proliferation by Modulating Stemness-Related Genes

**Figure 4:**
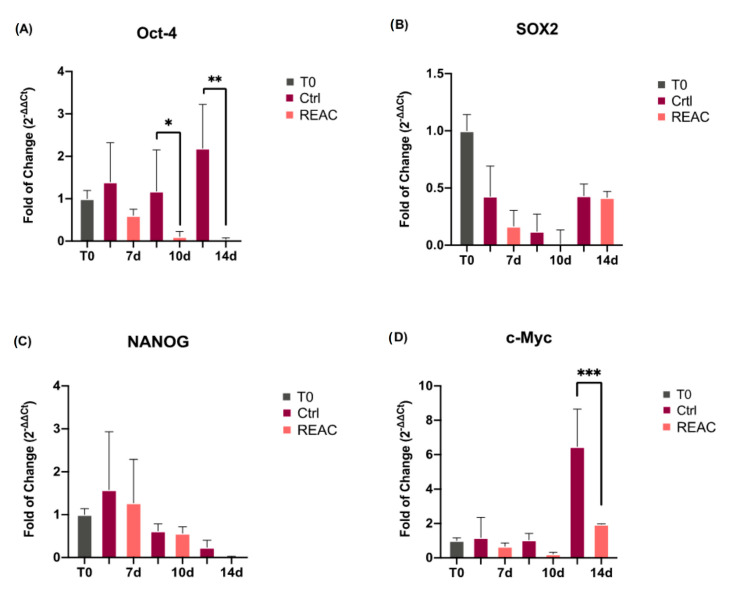
Effect of REAC TO-RGN treatment in modulating the level of expression of Oct-4 (Panel (**A**)), SOX2 (Panel (**B**)), NANOG (Panel (**C**)) and c-Myc (Panel (**D**)) in MCF7 either exposed or not to REAC TO-RGN treatment, after 7, 10 and 14 days in culture, as compared to control untreated cells. For each timepoint, the mRNA levels for each gene were normalized to GAPDH, and expressed as the fold of change (2^−∆∆Ct^) in the mRNA levels observed in control untreated cells at time 0 (before starting treatment), defined as 1. Data are expressed as mean ± SD relative to the control (* *p*  ≤  0.05; ** *p*  ≤  0.01; *** *p*  ≤  0.001).

Figure 4 shows the levels of expression of stemness-related genes, implicated in the progression and maintenance of cancer cells. Oct-4, Sox2 NANOG and c-Myc mRNA can be detected in MCF7 (Figure 4). Gene expression analysis was performed in MCF7 either treated or not with REAC TO-RGN treatment, after 7, 10 and 14 days in culture. As shown in Figure 4, the mRNA levels of Oct-4 (Panel A), Sox2 (Panel B), NANOG (Panel C) and c-Myc (Panel D) decreased in cells exposed to REAC TO-RGN treatment for each time point analyzed, reaching a statistically significant downregulation, especially at the end of 14 days in culture, as compared to control untreated cells. 

### 3.3. REAC TO-RGN Treatment Regulates Cell Cycle Progression 

**Figure 5:**
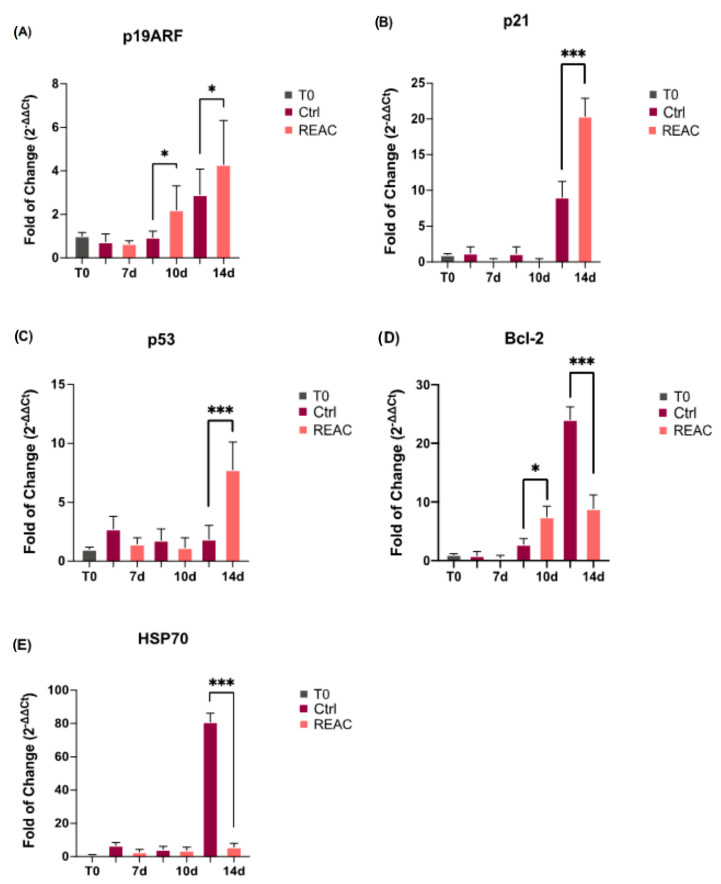
Effect of REAC TO-RGN treatment in modulating the level of expression of p19 (Panel (**A**)), p21 (Panel (**B**)), p53 (Panel (**C**)), Bcl-2 (Panel (**D**)) and HSP70 (Panel (**E**)) in MCF7 either exposed or not to REAC TO-RGN treatment, after 7, 10 and 14 days in culture, as compared to control untreated cells. For each timepoint, the mRNA levels for each gene were normalized to GAPDH and expressed as the fold of change (2^−∆∆Ct^) of the mRNA levels observed in control untreated cells at time 0 (before starting treatment), defined as 1. Data are expressed as mean ± SD relative to the control (* *p*  ≤  0.05; *** *p*  ≤  0.001).

Figure 5 shows the mRNA levels of p19ARF (Panel A), p21 (Panel B), and p53 (Panel C) in MCF7 either treated or not with REAC, after 7, 10 and 14 days in culture. The REAC TO-RGN treatment modulates p19ARF, p21, and p53 expression, a set of major cell cycle inhibitors and senescence-related genes, by determining their significantly upregulation, especially after 14 days in culture, as compared to control untreated cells. 

**Figure 6:**
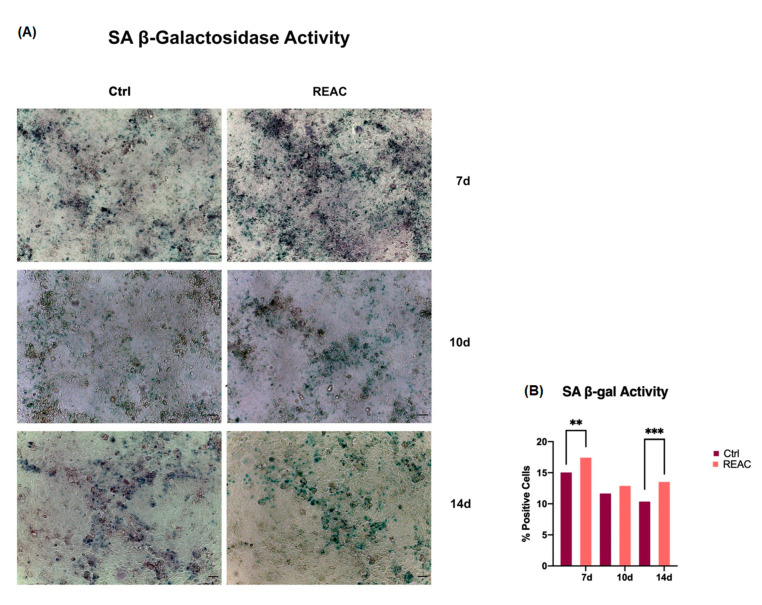
(**A**) Senescence-associated β-galactosidase activity. Cells were cultured for a period of 7, 10 or 14 days, as described above. Figures of cells exposed to REAC TO-RGN treatment were compared to control untreated cells (Ctrl). Magnification 10×. Scale bar = 100 μm. (**B**) The number of blue positive cells was calculated using ImageJ. The percentage of SA-β-Gal-positive cells was calculated as the number of positive cells divided by the total number of cells counted, using an image software analysis (ImageJ). Data are expressed as mean ± SD relative to the control (** *p*  ≤  0.01; *** *p*  ≤  0.001).

The gene expression analysis of p19, p21 and p53 were further inferred by β-galactosidase staining assay (Figure 6). 

Bcl-2, acting as an antiapoptotic genes, shows an opposite trend (Panel D). Its expression is significantly downregulated when MCF7 are exposed to a longer REAC TO-RGN treatment (14 days), as compared to control untreated cells, as well as HSP70, whose expression is significantly inhibited after 14 days in culture (Panel E). 

### 3.4. REAC TO-RGN Treatment Induces Cell Senescence 

Figure 6 shows the results from the β-galactosidase staining assay in MCF7 after 7, 10 and 14 days in culture. The colorimetric assay revealed that REAC significantly induces MCF7 senescence, as compared to control untreated cells (Panel A). The number of blue stained senescent cells was significantly increased after REAC TO-RGN treatment (Panel B). 

### 3.5. REAC TO-RGN Treatment Counteracts Tumor Progression Acting on DKK1 and SFRP1 Secretion

**Figure 7:**
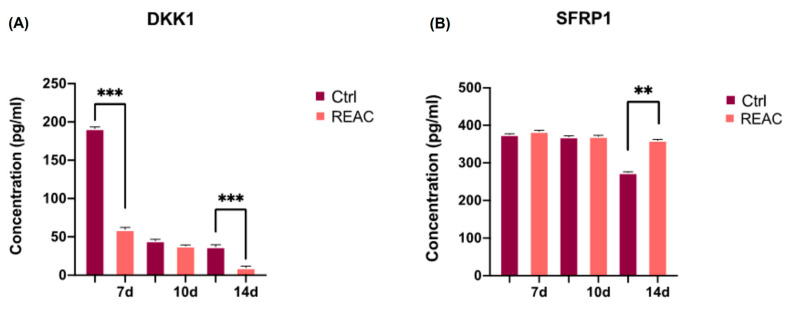
DKK1 and SFRP1 quantification by ELISA. The concentrations of DKK1 (**A**) and SFRP1 (**B**) were measured after 7, 10, and 14 days in supernatants of MCF7 either exposed or not to REAC TO-RGN treatment. Data are expressed as mean ± SD relative to the control (mean ± SD) (** *p* ≤ 0.01; *** *p*  ≤  0.001).

The concentration of DKK1 and SFRP1 secreted by MCF7 was evaluated by ELISA (Figure 7) in the supernatants of cells after 7, 10, and 14 days in culture. DKK1 was significantly downregulated in MCF7 exposed to REAC TO-RGN treatment even after 7 days in culture, as compared to control untreated cells (Panel A), and up to 14 days. SFRP1 also achieved the highest significant difference at the end of 14 days, being significantly increased in the supernatants of MCF7 exposed to REAC TO-RGN treatment (Panel B). 

### 3.6. REAC TO-RGN Treatment Inhibits Cell Proliferation by Inducing Autophagy

**Figure 8:**
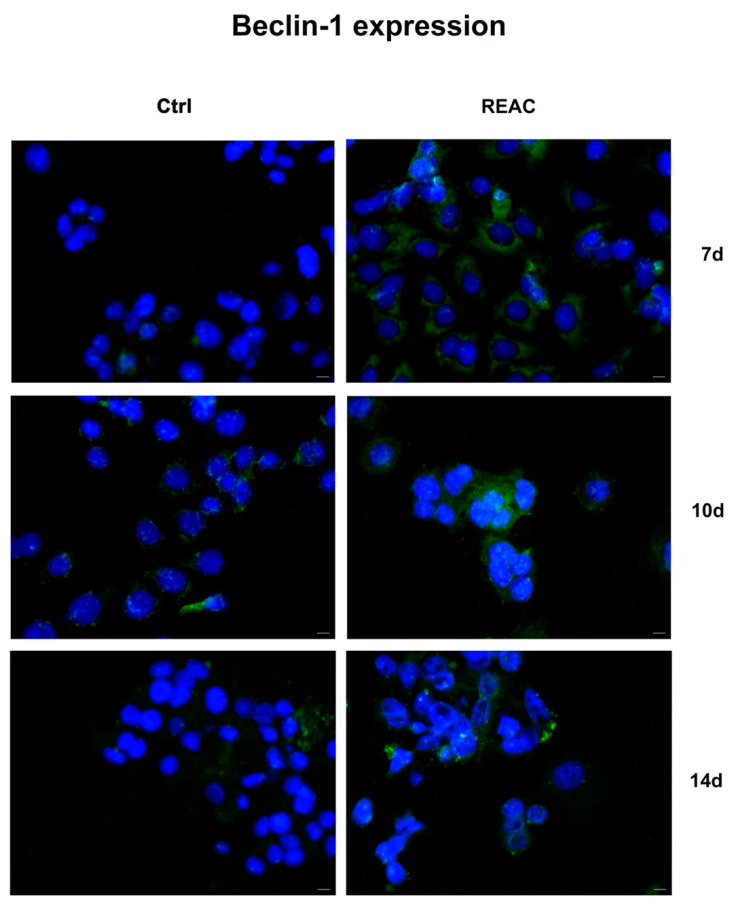
Analysis of Beclin-1 expression. Immunofluorescence analysis of the expression of Beclin-1 was assessed in cells cultured for a total of 7, 10 or 14 days, as above described. Figures of cells exposed to REAC TO-RGN treatment were compared to control untreated cells (Ctrl). Magnification 40×. Scale bar = 40 μm. The figures are representative of different independent experiments. Nuclei are labelled with 4,6-diamidino-2-phenylindole (DAPI, blue).

**Figure 9:**
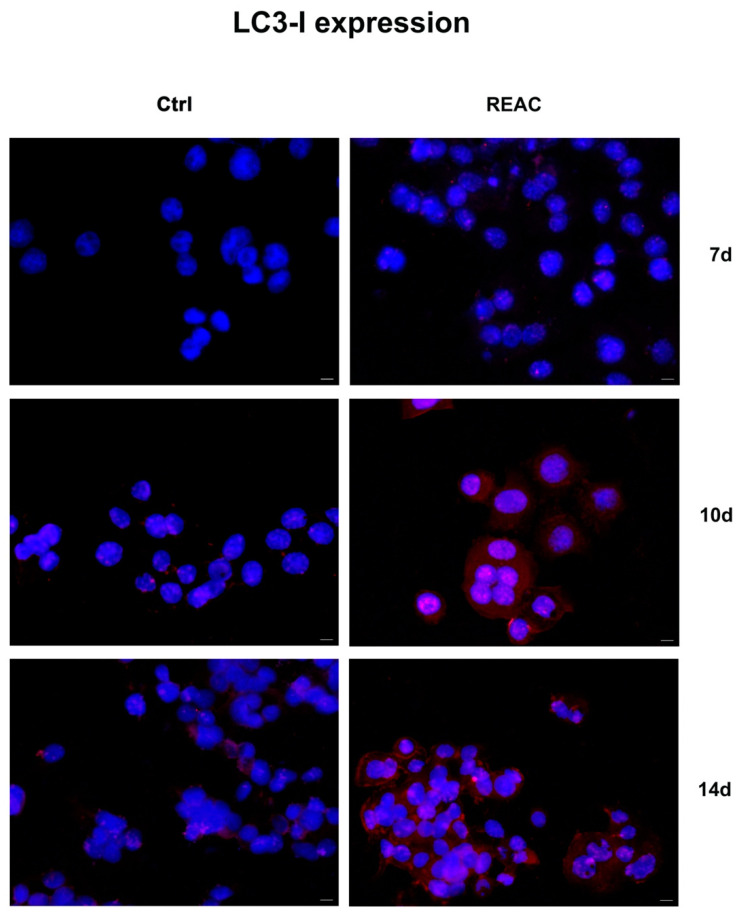
Analysis of LC3-I expression. Immunofluorescence analysis of the expression of LC3-I was assessed in cells cultured for a total of 7, 10 or 14 days, as above described. Figures of cells exposed to REAC TO-RGN treatment were compared to control untreated cells (Ctrl). Magnification 40×. Scale bar = 40 μm. The figures are representative of different independent experiments. Nuclei are labelled with 4,6-diamidino-2-phenylindole (DAPI, blue).

Immunofluorescence analysis shows the expression of autophagy-related markers, Beclin-1 and LC3-I, involved in the early stages of autophagy and in autophagosome formation, respectively. As shown in Figure 8, REAC TO-RGN treatment significantly induces Beclin-1 expression in MCF7 after 7 days in culture, as compared to control untreated cells. Its expression in treated MCF7 gradually decreases after 14 days, while LC3-I expression increases, as compared to untreated controls (Figure 9). 

## 4. Discussion

Precision medicine is a rapidly evolving field that aims to provide personalized treatment options to patients based on their individual genetic, environmental, and lifestyle factors. In recent years, there has been increasing interest in endogenous bioelectricity as a potential avenue for developing targeted and personalized therapies for cancer patients [10,42]. Endogenous bioelectricity refers to the bioelectric fields and signals produced by cells due to ion fluxes, which play a crucial role in cellular behavior, including cancer cell proliferation and metastasis [17]. V_mem_ [6] thus functions as an interface between chemical and mechanical signals, creating an electrical gradient between cells which, in turn, activates voltage-sensitive channels [4]. One of the ways in which carcinogenesis occurs is the disruption of electrical gradients, or the mechanisms by which they are sensed by cells. 

Cancerous tissues are generally depolarized relative to non-proliferative cells, and the cellular tumor microenvironment (TME) also affects bioelectric regulation and cell proliferation [43]. On the other hand, the role of electric fields in tumor processes, although it has been strongly established in recent decades, needs further investigation.

In this study, we used the REAC TO-RGN endogenous bioelectric manipulation treatment to reprogram the MCF7 breast cancer cell line. The results of the study demonstrated that the REAC TO-RGN treatment significantly reduced the viability and proliferation of MCF7 cells compared to control untreated cells. Additionally, the treatment altered the morphology of cancer cells, making them less aggressive. The study also found that the treatment modulated stemness-related genes, such as Oct-4, Sox2, and NANOG, plays a crucial role in the development and malignant progression of different types of tumors. Higher levels of Oct-4, Sox2, and NANOG were identified in MCF7 and MDA-MB-231 cells by previous authors [44]. REAC TO-RGN endogenous bioelectric manipulation was able to decrease the expression of all these markers, as well as of c-Myc. Overexpression of c-Myc leads to the onset and development of breast cancer. This transcription factor is a critical regulator of the TME, being involved in stromal cell growth and angiogenesis [45]. MCF7 exposed to REAC TO-RGN showed a significant downregulation of these markers, suggesting the ability of the technology to influence the cellular microenvironment and cell–microenvironment interactions. Moreover, REAC TO-RGN upregulated the expression of p19ARF, p21, and p53 genes, which are major cell cycle inhibitors, and senescence-related genes, also increasing the expression of autophagy-related markers Beclin-1 and LC3-I in treated cells, counteracting metastasis progression. 

These findings suggest that REAC TO-RGN endogenous bioelectric manipulation may represent a promising therapeutic strategy for cancer treatment. The ability to manipulate endogenous bioelectricity provides a non-invasive and targeted approach to cancer treatment that has the potential to improve patient outcomes. Numerous in vivo studies demonstrate how the manipulation of endogenous bioelectrical activity through specific treatments of the REAC technology are effective for the treatment of various muscle or nervous system disorders. The results of this study also have implications for the development of new therapeutic strategies for cancer treatment.

However, further research is needed to fully understand the mechanisms underlying the effects of REAC TO-RGN endogenous bioelectric manipulation on cancer cells, and to identify the optimal treatment conditions for different types of cancer.

## 5. Conclusions

In this study, we investigated the therapeutic potential of REAC TO-RGN treatment in inhibiting cancer cell proliferation and modulating stemness-related genes. Our findings demonstrate that REAC TO-RGN treatment can reprogram the bioelectric activity of cancer cells, leading to reduced cell viability and proliferation, and modulation of stem-ness-related genes. 

Additionally, we report that REAC TO-RGN treatment induces autophagy, a process in which cells degrade and recycle their own components, contributing to the observed reduction in cell proliferation. 

These results provide evidence of the potential therapeutic efficacy of REAC TO-RGN therapy in cancer treatment, and underscore the importance of understanding the role of bioelectric activity in cancer cell behavior.

The potential of endogenous bioelectricity as a therapeutic strategy for cancer is still being explored, and further research is needed to fully understand the mechanisms underlying its effects. However, the promising results of this study suggest that REAC TO-RGN endogenous bioelectric manipulation treatments may represent a future technological approach to precision medicine in the fight against cancer pathologies. 

## Figures and Tables

**Table 1:** Primer sequences.

Primer Name	Forward	Reverse
hGAPDH	GAGTCAACGGAATTTGGTCGT	GACAAGCTTCCCGTTCTCAG
Oct-4	GAGGAGTCCCAGGCAATCAA	CATCGGCCTGTGTATATCCC
Sox2	CCGTTCATGTAGGTCTCGGAGCTG	CAACGGCAGCTACAGCTAGATGC
NANOG	CATGAGTGTGGATCCAGCT	CCTGAATAAGCAGATCCAT
c-Myc	TGAGGAGACACCGCCCAC	CAACATCGATTTCTTCCTCATCTTC
p19ARF	GCCTTCGGCTGACTGGCTGG	TCGTCCTCCAGAGTCGCCCG
p21	CAAAGGCCCGCTCTACATCTT	AGGAACCTCTCATTCACCCGA
p53	TGGCCTTGAAACCACCTTTT	AACTACCAACCCACCAGCCAA
Bcl-2	TCGCTACCGTCGTGACTTC	AAACAGAGGTCGCATGCTG
HSP70	CACAGCGACGTAGCAGCTCT	ATGTCGGTGGTGGGCATAGA

**Table 2:** Cell viability and dimension of control and REAC-treated cells.

	T0	7 Days		10 Days		14 Days	
		Ctrl	REAC TO-RGN	Ctrl	REAC TO-RGN	Ctrl	REAC TO-RGN
Viability	100%	96.7%	91.9%	100%	96.7%	98.9%	83.6%
Dimension	12.1 μm	15.2 μm	12.3 μm	11.7 μm	10.6 μm	13.8 μm	11.9 μm

## Data Availability

Data is contained within the article.

## References

[1] Levin M., Pezzulo G., Finkelstein J.M. (2017). Endogenous Bioelectric Signaling Networks: Exploiting Voltage Gradients for Control of Growth and Form. Annu. Rev. Biomed. Eng..

[2] Levin M., Martyniuk C.J. (2018). The bioelectric code: An ancient computational medium for dynamic control of growth and form. Biosystems.

[3] Levin M. (2012). Molecular bioelectricity in developmental biology: New tools and recent discoveries: Control of cell behavior and pattern formation by transmembrane potential gradients. BioEssays.

[4] Levin M. (2014). Molecular bioelectricity: How endogenous voltage potentials control cell behavior and instruct pattern regulation in vivo. Mol. Biol. Cell.

[5] Adams D.S., Levin M. (2012). Endogenous voltage gradients as mediators of cell-cell communication: Strategies for investigating bioelectrical signals during pattern formation. Cell Tissue Res..

[6] Bhavsar M.B., Cato G., Hauschild A., Leppik L., Oliveira K.M.C., Eischen-Loges M.J., Barker J.H. (2019). Membrane potential (V_mem_) measurements during mesenchymal stem cell (MSC) proliferation and osteogenic differentiation. PeerJ.

[7] Levin M., Stevenson C.G. (2012). Regulation of Cell Behavior and Tissue Patterning by Bioelectrical Signals: Challenges and Opportunities for Biomedical Engineering. Annu. Rev. Biomed. Eng..

[8] Wang E.-T., Zhao M. (2010). Regulation of tissue repair and regeneration by electric fields. Chin. J. Traumatol..

[9] Sundelacruz S., Levin M., Kaplan D.L. (2008). Membrane Potential Controls Adipogenic and Osteogenic Differentiation of Mesenchymal Stem Cells. PLoS ONE.

[10] Chernet M.L.B. (2014). Endogenous Voltage Potentials and the Microenvironment: Bioelectric Signals that Reveal, Induce and Normalize Cancer. J. Clin. Exp. Oncol..

[11] Djamgoz M.B.A., Coombes R.C., Schwab A. (2014). Ion transport and cancer: From initiation to metastasis. Philos. Trans. R. Soc. B: Biol. Sci..

[12] Arcangeli A., Crociani O., Lastraioli E., Masi A., Pillozzi S., Becchetti A. (2009). Targeting Ion Channels in Cancer: A Novel Frontier in Antineoplastic Therapy. Curr. Med. Chem..

[13] Tuszynski J., Tilli T.M., Levin M. (2017). Ion Channel and Neurotransmitter Modulators as Electroceutical Approaches to the Control of Cancer. Curr. Pharm. Des..

[14] Pullar C.E. (2016). The Physiology of Bioelectricity in Development, Tissue Regeneration and Cancer.

[15] Levin M., Selberg J., Rolandi M. (2019). Endogenous Bioelectrics in Development, Cancer, and Regeneration: Drugs and Bioelectronic Devices as Electroceuticals for Regenerative Medicine. iScience.

[16] Lobikin M., Chernet B., Lobo D., Levin M. (2012). Resting potential, oncogene-induced tumorigenesis, and metastasis: The bioelectric basis of cancer in vivo. Phys. Biol..

[17] Payne S.L., Levin M., Oudin M.J. (2019). Bioelectric Control of Metastasis in Solid Tumors. Bioelectricity.

[18] Silver B.B., Nelson C.M. (2018). The Bioelectric Code: Reprogramming Cancer and Aging from the Interface of Mechanical and Chemical Microenvironments. Front. Cell Dev. Biol..

[19] Berzingi S., Newman M., Yu H.-G. (2016). Altering bioelectricity on inhibition of human breast cancer cells. Cancer Cell Int..

[20] Yu H.-G., McLaughlin S., Newman M., Brundage K., Ammer A., Martin K., Coad J. (2017). Altering calcium influx for selective destruction of breast tumor. BMC Cancer.

[21] Maioli M., Rinaldi S., Migheli R., Pigliaru G., Rocchitta G., Santaniello S., Basoli V., Castagna A., Fontani V., Ventura C. (2015). Neurological morphofunctional differentiation induced by REAC technology in PC12. A neuro protective model for Parkinson’s disease. Sci. Rep..

[22] Maioli M., Rinaldi S., Santaniello S., Castagna A., Pigliaru G., Delitala A., Bianchi F., Tremolada C., Fontani V., Ventura C. (2014). Radioelectric Asymmetric Conveyed Fields and Human Adipose-Derived Stem Cells Obtained with a Nonenzymatic Method and Device: A Novel Approach to Multipotency. Cell Transplant..

[23] Maioli M., Rinaldi S., Santaniello S., Castagna A., Pigliaru G., Gualini S., Cavallini C., Fontani V., Ventura C. (2013). Radio Electric Conveyed Fields Directly Reprogram Human Dermal Skin Fibroblasts toward Cardiac, Neuronal, and Skeletal Muscle-Like Lineages. Cell Transplant..

[24] Maioli M., Rinaldi S., Santaniello S., Castagna A., Pigliaru G., Gualini S., Fontani V., Ventura C. (2012). Radiofrequency Energy Loop Primes Cardiac, Neuronal, and Skeletal Muscle Differentiation in Mouse Embryonic Stem Cells: A New Tool for Improving Tissue Regeneration. Cell Transplant..

[25] Maioli M., Rinaldi S., Cruciani S., Necas A., Fontani V., Corda G., Santaniello S., Rinaldi A., Barcessat A.P., Necasova A. (2022). Antisenescence Effect of REAC Biomodulation to Counteract the Evolution of Myelodysplastic Syndrome. Physiol. Res..

[26] Maioli M., Rinaldi S., Pigliaru G., Santaniello S., Basoli V., Castagna A., Fontani V., Ventura C. (2016). REAC technology and hyaluron synthase 2, an interesting network to slow down stem cell senescence. Sci. Rep..

[27] Maioli M., Rinaldi S., Santaniello S., Castagna A., Pigliaru G., Delitala A., Margotti M.L., Bagella L., Fontani V., Ventura C. (2013). Anti-senescence efficacy of radio-electric asymmetric conveyer technology. Age.

[28] Rinaldi S., Maioli M., Pigliaru G., Castagna A., Santaniello S., Basoli V., Fontani V., Ventura C. (2014). Stem cell senescence. Effects of REAC technology on telomerase-independent and telomerase-dependent pathways. Sci. Rep..

[29] Van Schaijik B., Davis P.F., Wickremesekera A.C., Tan S.T., Itinteang T. (2017). Subcellular localisation of the stem cell markers OCT4, SOX2, NANOG, KLF4 and c-MYC in cancer: A review. J. Clin. Pathol..

[30] Casey S.C., Baylot V., Felsher D.W. (2018). The MYC oncogene is a global regulator of the immune response. Blood.

[31] Al Bitar S., Gali-Muhtasib H. (2019). The Role of the Cyclin Dependent Kinase Inhibitor p21cip1/waf1 in Targeting Cancer: Molecular Mechanisms and Novel Therapeutics. Cancers.

[32] Chen J. (2016). The Cell-Cycle Arrest and Apoptotic Functions of p53 in Tumor Initiation and Progression. Cold Spring Harb. Perspect. Med..

[33] Setroikromo R., Wierenga P., van Waarde M., Brunsting J., Vellenga E., Kampinga H. (2007). Heat shock proteins and Bcl-2 expression and function in relation to the differential hyperthermic sensitivity between leukemic and normal hematopoietic cells. Cell Stress Chaperon.

[34] Blackiston D.J., McLaughlin K.A., Levin M. (2009). Bioelectric controls of cell proliferation: Ion channels, membrane voltage and the cell cycle. Cell Cycle.

[35] Parfitt D.-E., Zernicka-Goetz M. (2010). Epigenetic Modification Affecting Expression of Cell Polarity and Cell Fate Genes to Regulate Lineage Specification in the Early Mouse Embryo. Mol. Biol. Cell.

[36] Rinaldi S., Fontani V., Castagna A., Lotti M., Ventura C., Maioli M., Santaniello S., Pigliaru G., Carta A., Gualini S. (2012). Regenerative treatment using a radioelectric asymmetric conveyor as a novel tool in antiaging medicine: An in vitro beta-galactosidase study. Clin. Interv. Aging.

[37] Rinaldi S., Collodel G., Fioravanti A., Pascarelli N.A., Fontani V., Maioli M., Santaniello S., Pigliaru G., Castagna A., Moretti E. (2013). Effects of regenerative radioelectric asymmetric conveyer treatment on human normal and osteoarthritic chondrocytes exposed to IL-1β. A biochemical and morphological study. Clin. Interv. Aging.

[38] Rinaldi S., Rinaldi C., Fontani V. (2022). Regenerative Radio Electric Asymmetric Conveyer Treatment in Generalized Cerebral and Cerebellar Atrophy to Improve Motor Control: A Case Report. Cureus.

[39] Castagna A., Fontani V., Rinaldi S. (2022). Radio Electric Asymmetric Conveyer Reparative Effects on Muscle Injuries: A Report of Two Cases. Cureus.

[40] Fontani V., Pereira J.A.C., Rinaldi S. (2022). Radio Electric Asymmetric Conveyer Tissue Reparative Treatment on Post-surgical Breast Skin Necrosis. A Report of Four Cases. Cureus.

[41] Cruciani S., Garroni G., Pala R., Barcessat A.R.P., Facchin F., Ventura C., Fozza C., Maioli M. (2022). Melatonin finely tunes proliferation and senescence in hematopoietic stem cells. Eur. J. Cell Biol..

[42] Zhu K., Hum N.R., Reid B., Sun Q., Loots G.G., Zhao M. (2020). Electric Fields at Breast Cancer and Cancer Cell Collective Galvanotaxis. Sci. Rep..

[43] Sheth M., Esfandiari L. (2022). Bioelectric Dysregulation in Cancer Initiation, Promotion, and Progression. Front. Oncol..

[44] Ling G.-Q., Chen D.-B., Wang B.-Q., Zhang L.-S. (2012). Expression of the pluripotency markers Oct3/4, Nanog and Sox2 in human breast cancer cell lines. Oncol. Lett..

[45] Gao F.-Y., Li X.-T., Xu K., Wang R.-T., Guan X.-X. (2023). c-MYC mediates the crosstalk between breast cancer cells and tumor microenvironment. Cell Commun. Signal..

